# Systematic Analysis of Functionally Related Gene Clusters in the Opportunistic Pathogen, *Candida albicans*

**DOI:** 10.3390/microorganisms9020276

**Published:** 2021-01-28

**Authors:** Sarah Asfare, Reem Eldabagh, Khizar Siddiqui, Bharvi Patel, Diellza Kaba, Julie Mullane, Umar Siddiqui, James T. Arnone

**Affiliations:** Department of Biology, William Paterson University, Wayne, NJ 07470, USA; asfares@student.wpunj.edu (S.A.); eldabaghr@wpunj.edu (R.E.); siddiquik@student.wpunj.edu (K.S.); patelb35@wpunj.edu (B.P.); kabad1@student.wpunj.edu (D.K.); mullanej1@student.wpunj.edu (J.M.); siddiquiu1@student.wpunj.edu (U.S.)

**Keywords:** *Candida albicans*, functional clustering, genomic organization, gene regulation, adjacent gene co-expression, stress response

## Abstract

The proper balance of gene expression is essential for cellular health, organismal development, and maintaining homeostasis. In response to complex internal and external signals, the cell needs to modulate gene expression to maintain proteostasis and establish cellular identity within its niche. On a genome level, single-celled prokaryotic microbes display clustering of co-expressed genes that are regulated as a polycistronic RNA. This phenomenon is largely absent from eukaryotic microbes, although there is extensive clustering of co-expressed genes as functional pairs spread throughout the genome in *Saccharomyces cerevisiae*. While initial analysis demonstrated conservation of clustering in divergent fungal lineages, a comprehensive analysis has yet to be performed. Here we report on the prevalence, conservation, and significance of the functional clustering of co-regulated genes within the opportunistic human pathogen, *Candida albicans*. Our analysis reveals that there is extensive clustering within this organism—although the identity of the gene pairs is unique compared with those found in *S. cerevisiae*—indicating that this genomic arrangement evolved after these microbes diverged evolutionarily, rather than being the result of an ancestral arrangement. We report a clustered arrangement in gene families that participate in diverse molecular functions and are not the result of a divergent orientation with a shared promoter. This arrangement coordinates the transcription of the clustered genes to their neighboring genes, with the clusters congregating to genomic loci that are conducive to transcriptional regulation at a distance.

## 1. Introduction

Many *Candida* species are opportunistic pathogens, with cells undergoing profound transcriptional changes during the transition into a pathogenic form. These pathogens are a major cause of morbidity and mortality across the world and represent a significant public health threat [1,2]. When pathogenic, *Candida* species can manifest as: candidemia, in the formation of biofilms, urinary tract infections, vulvovaginal infections, thrush, and associated invasive candidiasis [3,4,5,6,7]. There are a finite number of treatments available, including azoles, flucytosine and echinocandin drugs [8]. Unfortunately, multidrug resistance to available options is on the rise and driven in part by genomic instability and a mutator phenotype [8,9,10]. Further complicating the situation is the emergence of novel, more pathogenic species, such as *C. auris*, highlighting the need for a more complete understanding of the biology of these species. Of particular importance is the genomic organization, transcriptional regulation, and regulatory mechanisms associated with the changes in the lifecycle of *Candida* strains—such as during the switch to infection and pathogenesis [11,12,13,14].

Proper gene regulation is essential to all organisms to establish cellular identity and maintain homeostasis [15,16,17,18,19]. All single-celled eukaryotic organisms, including *Candida albicans*, deal with the complexities of this response with multiple levels of gene expression regulation [20,21,22,23,24,25]. Multiple pathways recognize signals—both internal and external—and respond, ultimately converging to alter the transcriptome of the cell, triggering distinct gene expression programs and specific transcriptional signatures [26,27]. These changes are regulated at multiple levels by myriad mechanisms, ensuring adaptation to the environmental niche that the cell occupies [28,29,30]. In single-celled organisms, including yeasts, these changes are rapid and can result in alterations to the expression of thousands of genes simultaneously to ensure survival [28,31].

The regulatory circuitry that is required to survive in the host depends, in part, on chromatin remodeling and the production of chaperone proteins [32]. Biofilm formation depends on the activity of several transcription factors that target over one thousand genes [33,34]. An additional layer of transcriptional regulation is through genomic organization. The spatial positioning of functionally related, co-expressed gene families as clusters throughout the genome is a central organizing principal in the related *Ascomycete*, *Saccharomyces cerevisiae* [35,36]. This arrangement—the functional clustering of co-expressed genes that participate in a shared molecular process—helps to minimize the effects of stochastic gene expression and noise [36,37,38]. There are significant levels of genomic clustering observed in the ribosomal protein (RP) and rRNA and ribosome biosynthesis (RRB) gene families in *C. albicans*, and while the absolute levels of clustering present are comparable to those in *S. cerevisiae*, the identity of the clusters differs [35,39,40]. At present, a systematic analysis of the prevalence and significance of genomic organization on transcriptional coregulation has yet to be performed in *C. albicans*.

In this work, we systematically characterize the genomic organization of functionally related, co-expressed gene families into clusters throughout the genome of the fungal pathogen, *C. albicans*. We report that the genomic distribution of gene families into clusters is widespread in *C. albicans* and that this arrangement is extensively conserved within related *Candida* species. The co-localization of genes as functional clusters frequently results in tighter transcriptional co-expression for the clustered genes. Altogether, our work suggests that this organization represents an integral level of organization to facilitate and coordinate the co-expression of functionally related genes.

## 2. Materials and Methods

### 2.1. The Identification and Significance of Functional Clustering in Candida albicans

The prevalence and statistical probability of genomic arrangement was determined by accessing the membership of each gene family annotated by the gene ontology (GO) molecular process designations from the *Candida* Genome Database [39,40,41]. The global, genomic distribution for the thirty-eight gene families was subsequently determined by manual curation throughout the *Candida* haploid genome, as previously described [35]. Once the family membership, locations, and orientations were mapped, the significance of this genomic arrangement was determined by calculating the binomial probability for the arrangement as previously described [42].

### 2.2. The Conservation of the Functional Clusters across Divergent Candida Lineages

The conservation of the observed genomic distribution was determined by manual curation from the *Candida* gene order browser (CGOB) [43,44]. The conservation of syntenic relationships identified in *C. albicans* was explored in the following *Candida* and yeast strains: WO1, *C. dubliniensis*, *C. tropicalis*, *C. parapsilosis*, *C. metapsilosis*, *C. orthopsilosis*, *L. elongisporus*, *D. hansenii*, *S. stipitis*, *C. tenuis*, *S. passalidarum*, *M. guilliermondii*, *C. lusitaniae*, *C. auris*, and *S. cerevisiae*. Gene clusters were queried as pairings (to account for the partial conservation of larger clusters) and the relationships were defined as conserved if the syntenic context was identical within a divergent *Candida* genome. Heat maps were generated to facilitate visualization in a binary fashion, with each grouping defined as conserved (yellow) or not conserved (black). Genes were arranged by family membership and then by the extent of conservation within each family, omitting ‘Cell budding’, ‘Cell wall organization’, ‘Conjugation’, ‘Protein folding’, and the ‘Transposition’ gene families, as there were no identified functional clusters in *Candida*.

### 2.3. Calculation of the Transcriptional Relationship within Gene Families with the Pearson’s Correlation Coefficient

The average pairwise Pearson’s correlation coefficient (PCC) from gene expression data was calculated as previously described [42]. The PCC scores for the unpaired genes were calculated from the average of every possible pairing partner for every possible gene. *P* values were determined by bootstrapping with replacement by taking at least 10,000 random groupings of genes (the same size as the paired subset) and determining the average PCC score for that grouping.

### 2.4. Calculation of the Transcriptional Relationship within a Genomic Neighborhood Using the Spearman’s Correlation Coefficient

Determination of transcriptional coregulation at a distance was calculated by the Spearman’s correlation coefficient (SCC). The Spearman’s correlation coefficient was determined as described previously [45]. Calculations were performed using Python and the Pandas library. Values were plotted as a function of distance, using the genomic distance between transcriptional start sites for two genes.

### 2.5. Microarray and RNA-Sequencing Data Sets Used for Analysis

The datasets analyzed for gene expression analysis were accessed and downloaded from the Gene Expression Omnibus in the National Library of Medicine (https://www.ncbi.nlm.nih.gov/geo/). Conditions that induced a broad range of cellular transcriptional responses for analysis were selected for analysis. The microarray and RNA-sequencing gene expression datasets utilized were: the transcriptional changes in response to carbon shift from glucose to xylose (GSE50476), response to the anti-fungal thiosemicarbazone, NSC319726, (GSE106486), response to the sterol alkaloid tomatidine (GSE96965), and the response to mucin (GSE149196) [46,47,48].

## 3. Results

### 3.1. Functionally Related Genes Cluster throughout the Candida albicans Genome

The prevalence of the functional clustering in *Candida albicans* was determined for the thirty-eight gene families annotated by the Gene Ontology Consortium as having a shared Molecular Process [39]. The individual members of each family were accessed and the genomic location of every member was curated utilizing the Candida Genome Database to determine the occurrence of clusters within each family (Table 1) [41]. There were 38 gene families in our initial analysis, with the individual gene families varying in size from as small as four genes, as in the ‘Transposition’ gene family, up to 1500 genes, as in the ‘Regulation of biological process’ family. The broad scope of several of the larger gene families, including ‘Regulation of biological process’ and ‘Organelle organization’ (1053 members), served as controls—these families are non-specific umbrella terms that include multiple members from more specific families—and thus would not be expected to cluster to a significant degree. The composition of these groups in particular consist of members that are cross-listed under other, more specific ontology headings.

For completion of this analysis, we did not exclude any members throughout this phase of the project. A significant fraction of the queried gene families—twenty out of the thirty-eight families—exhibit a non-random distribution throughout the genome (utilizing a threshold for significance of *p* < 0.05). Eight families exhibit a highly significant distribution (*p* < 0.001): ‘Biofilm formation’, ‘Carbohydrate metabolic process’, ‘Cell adhesion’, ‘Cell cycle’, ‘Cytoskeleton organization’, ‘Generation of precursor metabolites and energy’, ‘Translation’, and ‘Vitamin metabolic process’ (Table 1). As expected, the largest gene families, that represented umbrella terms and are composed of genes from a variety of more specific molecular functional groups exhibited a random distribution using our statistical cutoff.

### 3.2. Functionally Clustered Genes Exhibit Tighter Transcriptional Coregulation within Their Gene Family

In order to determine the effect of clustering on the transcription within a co-regulated gene family, the Pearson’s correlation coefficient (PCC) was calculated throughout gene expression changes induced by a variety of growth and lifestyle conditions (complete conditions and datasets are listed in the Materials and Methods section). Microarray and RNA-sequencing (RNA-seq) gene expression datasets were accessed and the transcription profiles for each of the gene families that displayed a clustered organization were extracted (the five gene families that contained zero clusters among their membership were not included in these analyses).

The average pairwise PCC values for the entire gene family, the pairwise PCC values for the clustered members of the gene family, and the pairwise PCC values for the unclustered, or singleton, members of each gene family were calculated (Table 2). Our analysis shows that across all conditions, all gene families studied have a positive correlation, although there is significant variation between families—with the ‘Ribosome biogenesis’, ‘Nucleus organization’, ‘Translation’, and ‘RNA metabolic processes’ displaying the tightest transcriptional coordination. Using a cutoff of a difference in transcriptional coordination of ≥0.1, we identified six gene families where the clustered genes are more tightly co-expressed together and another ten families where there was a slight increase relative to the singleton family members. Surprisingly, eight families have a more significant correlation for the singletons rather than the clustered set of genes. One gene family, ‘Pseudohyphal growth’, demonstrated an anti-correlation that is characteristic of mutually exclusive gene expression, potentially by transcriptional interference.

### 3.3. Clustered Genes Do Not Exhibit a Bias for a Divergent Orientation

There are three potential orientations for clustered genes, which can be found in a divergent (←→), tandem (→→ or ←←), or convergent orientation (→←) with respect to their transcription start sites. Of the three possibilities, a divergent orientation provides a facile model for the coregulation of a gene cluster via the function of a shared bi-directional promoter. In order to ascertain if the gene clusters were biased towards this particular mechanism, the relative orientations for every functional cluster were determined and annotated (Table 3).

For this analysis, clusters that appeared under multiple ontological headings were eliminated, keeping only the most specific assigned molecular function. This reduced the number of genes in this analysis significantly—from a total of 2945 total clustered members to 1452 unique clusters spanning all 33 ontology categories. There is no observable bias towards a divergent orientation, with an approximately even split between divergent (←→), where there are 389 pairings found in this orientation, and convergent (→←), where there are 343 pairings found in this orientations. The only observable bias is towards a tandem (→→ or ←←) orientation, which is overrepresented relative to the other two possibilities with a total of 720 pairings in this orientation.

### 3.4. Clustered Grouping Are Highly Conserved among Closely Related Candida Species and Deteriorate with Greater Evolutionary Distance

**Figure 1:**
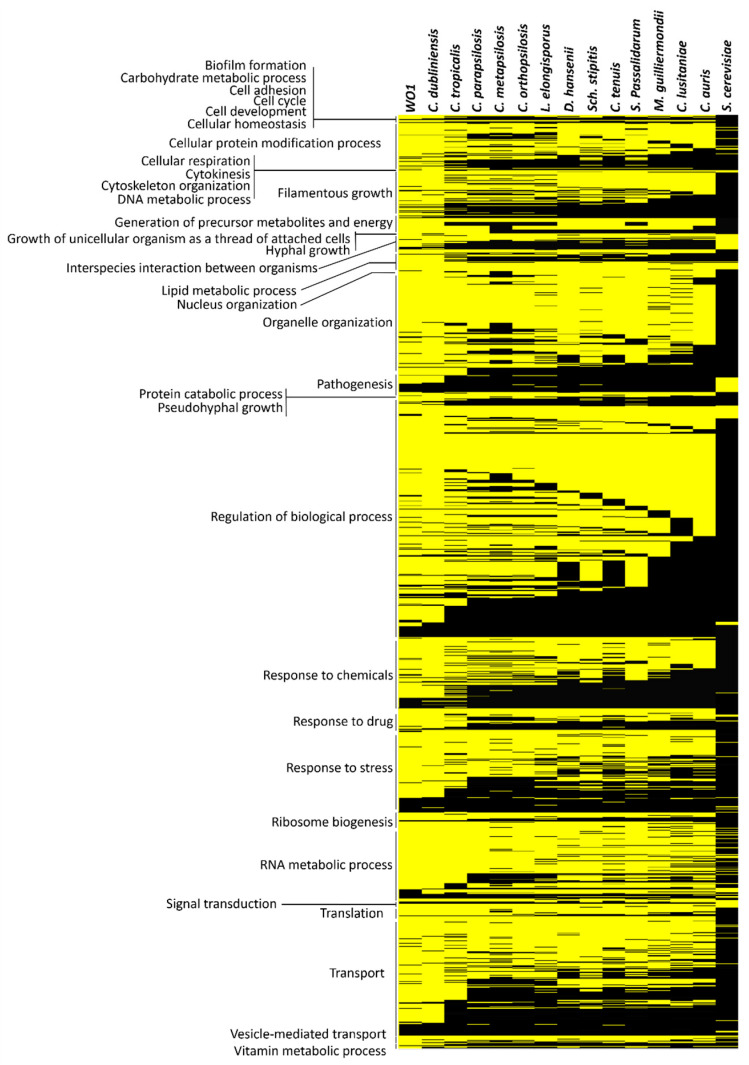
Conservation of functionally related clusters in divergent fungal lineages. The functionally clustered gene pairings from *Candida albicans* were analyzed for conservation of the pairings. The heat map depicts the conservation with either yellow (conservation of the pairing) or black (no conservation of the pairing).

To characterize the relationship between clustered genes within this particular clade, the conservation of the identity of clustered genes was determined across extant *Candida* lineages through the budding yeast, *Saccharomyces cerevisiae*. The unique set of clustered genes was individually queried from *Candida albicans* as a reference point and were assigned a binary score as either conserved or not conserved for this analysis. Analysis is performed in *C. dubliniensis*, *C. tropicalis*, *C. parapsilosis*, *C. metapsilosis*, *C. orthopsilosis*, *L. elongisporus*, *D. hansenii*, *S. stipitis*, *C. tenuis*, *S. passalidarum*, *M. guilliermondii*, *C. lusitaniae*, *C. auris*, and *S. cerevisiae*, and is visualized as a heat map (Figure 1). For simplicity, individual clusters are grouped by family and then by the extent of conservation within the family. The identity of clusters was highly conserved within the closely related *Candida* species, consistent with previous analysis performed across *Saccharomyces* lineages [42]. The conservation of clustered gene identity decreases as the evolutionary distance between *Candida albicans* and the fungal lineage for comparison increased, consistent with previous observations. The conservation eroded significantly when compared to the distantly related budding yeast, *S. cerevisiae*, indicating that the majority of clustered relationships most likely arose after the evolutionary divergence between these species.

### 3.5. Functionally Clustered Gene Sets Display a Higher Transcriptional Coregulation than Would Be Expected by Chance Groupings of Functionally Related Genes

**Figure 2:**
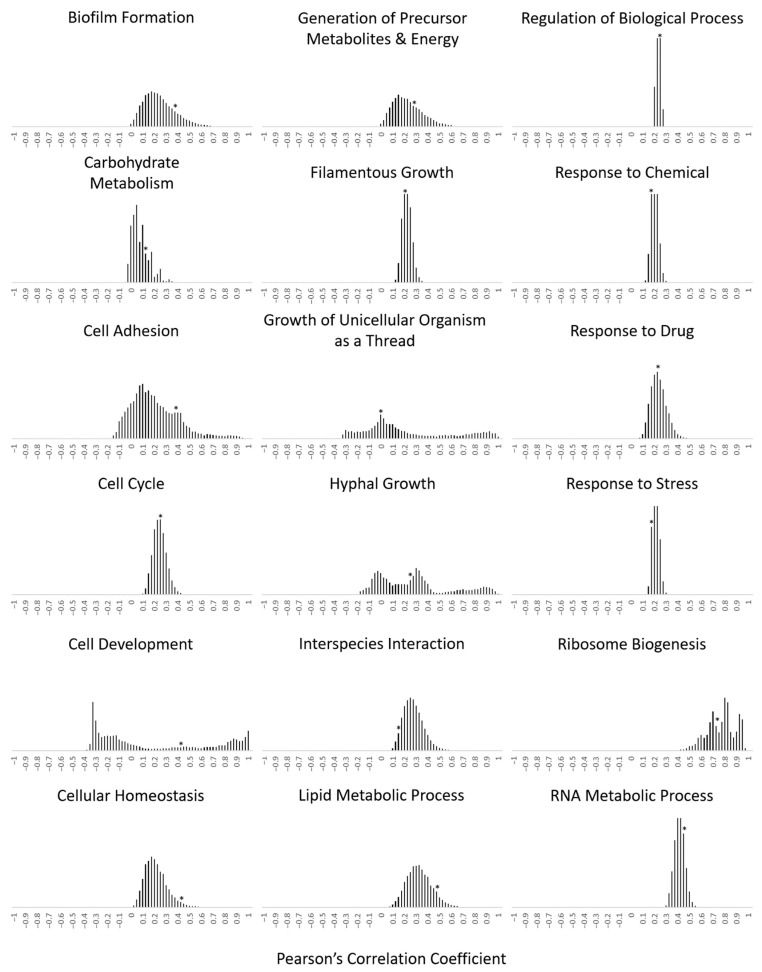

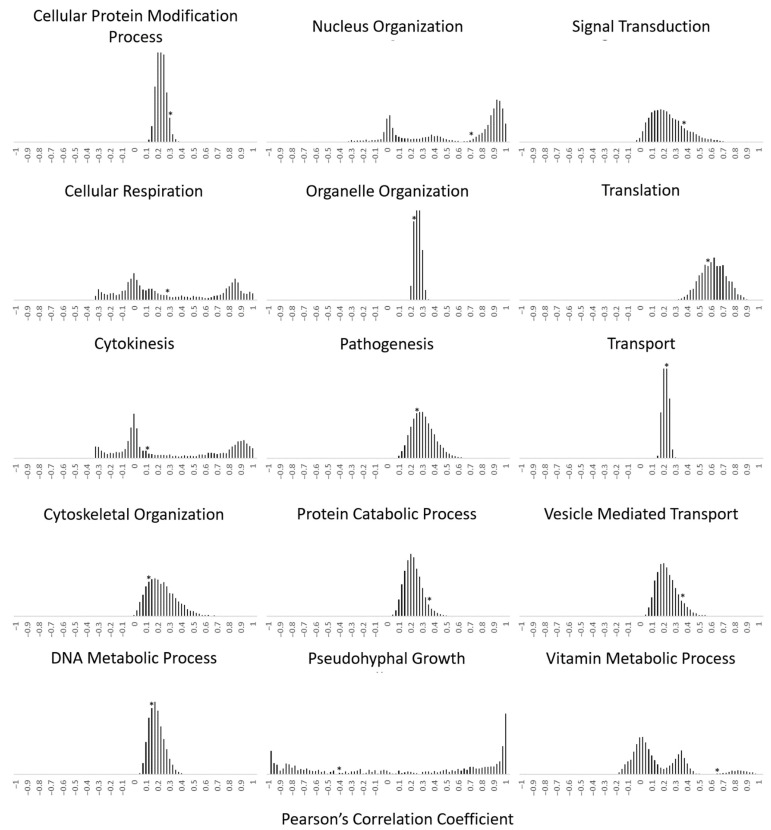
Many functionally clustered gene sets exhibit tighter transcriptional coregulation than random subgroupings of comparable sizes. Shown is the transcriptional correlation for every possible functional clustering arrangement that could have arisen through bootstrapping with replacement. The PCC was calculated for 10,000 iterations that represent every possible combination of clustering that could have evolved (of comparable size to the actual cluster for each set), and the frequency histograms are presented. The actual PCC for the clustered gene set within each family is marked with an asterisk.

Previous studies in yeasts have characterized the set of clustered genes as distinct from the many possible clustered combinations that could have arisen by evolutionary chance [42]. In order to test the transcriptional effect of clustering to that of all the genes that could have been grouped together, a bootstrapping analysis was performed with the actual grouping’s PCC denoted with an asterisk (Figure 2). By extending this type of analysis to the *Candida albicans* clustered genes set, we report a significant number of grouping combinations that have a tighter transcription profile than the actual paired set—even in some of the grouping classifications that contained the highest PCC values, such as ‘Ribosome biogenesis’. There is a correlation identified within the non-random clustered sets, where the ‘Biofilm formation’, ‘Carbohydrate metabolic process’, ‘Cell adhesion’, ‘Generation of precursor metabolites and energy’, ‘Lipid metabolic process’, ‘Protein catabolic process’, ‘RNA metabolic process’, and ‘Vitamin metabolic process’ all exhibit a higher than expected PCC by chance alone. The control sets (non-significant genomic distribution) that include the ‘Regulation of biological process’, ‘Response to drug’, and ‘Response to stress’ do not exhibit the same pattern. It is interesting that in all cases, the actual clustered sets all exhibit a higher PCC value than the least clustered members of the sets, in many cases bordering on the threshold for statistical significance. This is consistent with the role that *cis*- and *trans*-factors play in coordinating the transcription of each functional set of genes, with spatial positioning potentially reinforcing this process during the stress conditions explored in our analysis.

### 3.6. Functionally Related Genes Congregate to Transcriptionally Permissive Regions throughout the Genome

**Figure 3:**
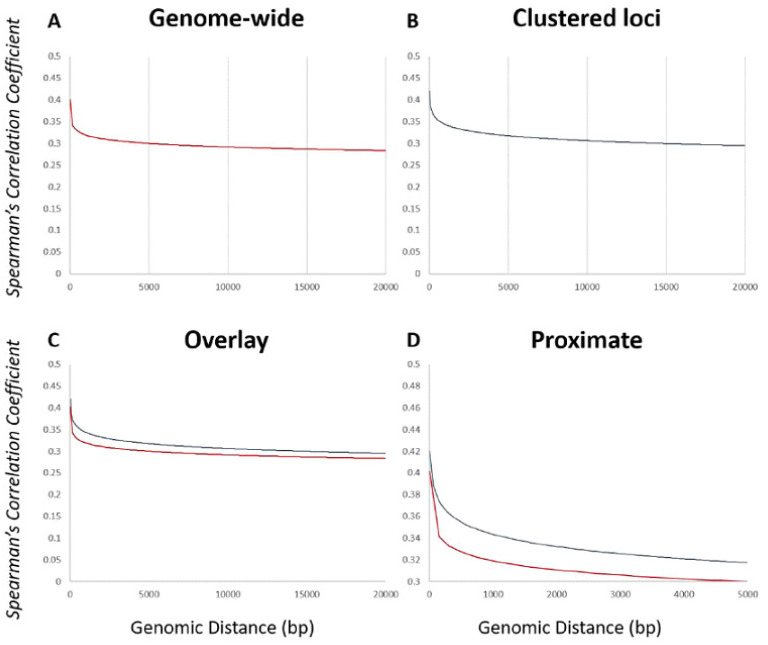
Functionally related genes congregate to transcriptionally permissive genomic loci. The Spearman’s correlation coefficient was calculated as a function of genomic distance globally (**A**) and for the functionally clustered gene set (**B**) and the logarithmic decay of best fit was determined for the datasets. These functions were overlaid (**C**,**D**) for clarity of comparison. The red trend line represents the genome-wide dataset and the blue represents the clustered functions.

Functionally related genes are clustered into genomic loci that exhibit a stronger influence on the expression of their neighboring genes in certain yeasts [45]. In order to determine if the *Candida albicans* functional clusters are found in loci with similar properties, the Spearman’s correlation coefficient (SCC) was calculated between each gene and their ten closest neighboring genes, which was then plotted as a function of genomic distance. A line of best fit was plotted over the data to facilitate interpretation. Our analysis focused on a global analysis, which was compared to an analysis of the clustered set of genes (Figure 3A,B). Consistent with observations seen across eukaryotic lineages, there is a global transcriptional similarity (positive SCC value) between all genes that decays as a function of distance (Figure 3A). The transcriptional similarity at loci where the clustered set of genes congregates displays a similar pattern, however at close genomic distances, there is a larger SCC value (Figure 3B). For ease of comparison, the two plots were superimposed on the same graph, scaled to view the global SCC and then zoomed into the proximate region alone (Figure 3C,D, respectively). This comparison confirms that the clustered loci are found in regions that exhibit a stronger transcriptional similarity as measured by the SCC than a typical genomic locus. The rate of decay is similar and approaches the same basal level that is observed across the conditions analyzed. One surprising observation was that the decay does not approach zero in our analysis; it approaches a value of approximately 0.3. This is most likely due to the nature of the conditions analyzed, which typically invoke a stress (or stress-like) response in this organism. As this is the case we believe that the positive value rather than a zero value at large genomic distances is an artifact of the data sets analyzed, rather than indicative of a global positive SCC value irrespective of genomic distance in this organism, and would not be predicted to be observed in cycling and unstressed cells populations.

## 4. Discussion

The clustering of functionally related gene families in the budding yeast, *S. cerevisiae*, is an organizational feature of the gene sets that contribute to ‘adjacent gene coregulation’ [49,50]. While initially recognized in the ‘Ribosomal protein’ and the ‘rRNA and ribosome biogenesis’ gene families, clustering has subsequently been identified in numerous gene families in *S. cerevisiae* [35,51]. Here, our analysis expands the characterization of the functional clustering of co-regulated genes to *Candida albicans* and related *Candida* lineages. We report that there is extensive gene clustering observed in functionally related, co-expressed gene families in *C. albicans*. This is conserved, at least in part, throughout related *Candida* lineages. The extent of the conservation of clustering correlates with evolutionary distance between species compared.

One surprising observation is that even when there are comparable levels of clustering within a co-regulated gene family, the identity of the clustered members differ between divergent fungi. Taking the ‘Ribosomal protein’, ‘rRNA and ribosome biogenesis’, and ‘Vitamin metabolic process’ gene families as examples, there are comparable levels of clustering observed in all three of these families in both *C. albicans* and in *S. cerevisiae*. The individual membership of each family is not conserved, as the gene members that comprise the functional clusters differ in each species. While this is consistent with previous observations, this present work expands upon this greatly, supporting a model where clustering is occurring in different species via mechanisms that converge at this arrangement [35,49]. This overlap of clustered genes is minimal when compared to *S. cerevisiae*—consistent with previous findings taken from that species—indicating that the majority of the clustered genes within each gene family arose independently within these two lineages [35].

When clustered together, the genes exhibit a coupled transcriptional correlation in many cases. These most likely result in a fitness advantage via the minimization of the stochastic influences on transcription. The mechanism that links transcription within clusters has yet to be decisively elucidated, however there is evidence supporting the role of chromatin remodelers mediating changes at the local level to modulated transcription within a genomic neighborhood [35]. While genes that are found in a divergent orientation oftentimes have bidirectional promoters, as seen in the histone protein coding genes, this does not appear to be a major factor in the regulation of clusters in *C. albicans* [52,53]. The fact that there is a disproportionate incidence of gene clusters found in a tandem orientation is surprising—there are incidences of tandem oriented pairs exhibiting mutually exclusive transcription patterns, as in *SER3* transcription and heat shock protein gene family [49,54,55]. This could explain the number of cases where there was a less positive PCC for clustered genes. Positive transcriptional regulation in this manner could be the result of bicistronic transcripts, which are under-characterized in yeasts. It would be fascinating to see if this mechanism is utilized to a significant degree in *Candida albicans*.

The benefit of clustering co-expressed genes together is two-fold. This eliminates the potential incidence of toxic intermediate compounds within biosynthetic pathways and it facilitates stoichiometric levels of proteins that are necessary in specific complexes—both of which confer a survival benefit and could serve as the selection mechanism for this organization [37,38]. We postulate that the drivers of this clustering arrangement are likely a combination of these two mechanisms. The ability to maintain proteostasis is vital, as orphan proteins that are not incorporated into a complex can induce proteotoxic stress and decrease viability, as seen in the RP family [38]. A clustered arrangement may help to buffer the stochastic noise inherent in gene expression and link the expression of genes to minimize this effect. This would explain why there are comparable levels of clustering in certain families, albeit with the different composite memberships that we observe. If two (or more) genes were clustered in a manner that provides a protective benefit by limiting toxic intermediate compounds within a pathway, we would potentially expect those genes to be clustered across a long evolutionary distance. The galactose and biotin biosynthetic pathways are two examples of these groupings, and indeed they are clustered together in such a fashion [36]. While beyond the scope of this work, it would be fascinating to see if the clusters that are conserved over longer distances do have this relationship in *Candida* species—such an analysis could potentially identify toxic factors during infectivity, and there are oftentimes detoxification mechanisms clustered with the toxin synthesizing enzymes to protect the cell [36].

## 5. Conclusions

Though it has been observed that genome-wide global transcriptional similarities have been observed in yeasts and other eukaryotes for a long time, the functional benefit to eukaryotes is still in the process of being fully understood [54,55]. The physical arrangement of genes along the chromosome coordinates transcription of functionally related genes at many loci. This allows for tighter, more efficient transcription of genes whose functions participate in a shared molecular process by the process of adjacent gene coregulation [49]. Alternatively, this configuration can facilitate mutually exclusive expression of select pairings via transcriptional interference. *Candida albicans* and related *Candida* lineages exhibit extensive genomic organization in this manner and this may play a significant role in organismal adaptation, survival, and pathogenesis.

## Figures and Tables

**Table 1:** Gene Clustering within a Shared Molecular Process in Candida albicans.

Molecular Process	Gene Family Size	Singletons	Clusters	*p*-Value
Biofilm formation	149	132	17	2.53 × 10^−4^
Carbohydrate metabolic process	191	168	23	8.19 × 10^−4^
Cell adhesion	68	61	7	2.94 × 10^−5^
Cell budding	46	46	0	n.s
Cell cycle	477	375	102	1.02 × 10^−4^
Cell development	113	110	3	6.03 × 10^−1^
Cell wall organization	160	160	0	n.s.
Cellular homeostasis	205	183	22	9.99 × 10^−3^
Cellular protein modification process	584	452	132	3.92 × 10^−3^
Cellular respiration	89	85	4	1.19 × 10^−1^
Conjugation	63	63	0	n.s.
Cytokinesis	91	87	4	1.36 × 10^−1^
Cytoskeleton organization	184	157	27	5.38 × 10^−6^
DNA metabolic process	344	290	54	3.24 × 10^−3^
Filamentous growth	607	470	138	9.55 × 10^−3^
Generation of precursor metabolites and energy	140	119	21	4.34 × 10^−7^
Growth of unicellular organism as a thread of attached cells	88	84	4	1.11 × 10^−1^
Hyphal growth	97	91	6	3.50 × 10^−2^
Interspecies interaction between organisms	343	300	43	1.58 × 10^−1^
Lipid metabolic process	286	249	37	1.44 × 10^−2^
Nucleus organization	56	52	4	3.68 × 10^−3^
Organelle organization	1053	682	371	6.68 × 10^−3^
Pathogenesis	275	239	36	7.33 × 10^−3^
Protein catabolic process	220	196	24	1.44 × 10^−2^
Protein folding	82	82	0	n.s.
Pseudohyphal growth	41	39	2	1.78 × 10^−2^
Regulation of biological process	1500	859	641	6.73 × 10^−1^
Response to chemical	804	591	213	1.19 × 10^−1^
Response to drug	406	343	63	5.53 × 10^−2^
Response to stress	860	614	246	6.24 × 10^−2^
Ribosome biogenesis	305	277	28	5.29 × 10^−2^
RNA metabolic process	774	551	221	1.23 × 10^−3^
Signal transduction	219	203	16	3.85 × 10^−1^
Translation	245	207	38	2.45 × 10^−5^
Transport	1060	698	362	5.45 × 10^−2^
Transposition	4	4	0	n.s.
Vesicle-mediated transport	320	290	30	6.62 × 10^−1^
Vitamin metabolic process	40	34	6	8.64 × 10^−7^

Candida haploid genome: 6066 total open reading frames. n.s.—not significant.

**Table 2:** Pearson’s Correlation Coefficient of Functionally Related Genes.

Molecular Process	Family	Singletons	Clustered Set
Biofilm formation	0.282	0.266	0.376
Carbohydrate metabolic process	0.212	0.224	0.101
Cell adhesion	0.274	0.283	0.370
Cell cycle	0.258	0.256	0.260
Cell development	0.216	0.215	0.433
Cellular homeostasis	0.283	0.266	0.429
Cellular protein modification process	0.289	0.276	0.331
Cellular respiration	0.246	0.250	0.293
Cytokinesis	0.299	0.300	0.100
Cytoskeleton organization	0.241	0.262	0.116
DNA metabolic process	0.242	0.260	0.148
Filamentous growth	0.281	0.295	0.237
Generation of precursor metabolites and energy	0.237	0.231	0.287
Growth of unicellular organism as a thread of attached cells	0.280	0.301	0.017
Hyphal growth	0.308	0.306	0.287
Interspecies interaction between organisms	0.348	0.368	0.182
Lipid metabolic process	0.345	0.327	0.481
Nucleus organization	0.540	0.525	0.772
Organelle organization	0.285	0.310	0.240
Pathogenesis	0.363	0.381	0.243
Protein catabolic process	0.328	0.329	0.352
Pseudohyphal growth	0.260	0.262	−0.438
Regulation of biological process	0.295	0.304	0.282
Response to chemical	0.269	0.296	0.196
Response to drug	0.290	0.298	0.253
Response to stress	0.316	0.302	0.229
Ribosome biogenesis	0.680	0.673	0.736
RNA metabolic process	0.416	0.415	0.470
Signal transduction	0.288	0.279	0.372
Translation	0.525	0.531	0.582
Transport	0.264	0.271	0.250
Vesicle-mediated transport	0.319	0.313	0.368
Vitamin metabolic process	0.258	0.227	0.641

**Table 3:** Orientation of Functional Clusters.

Molecular Process	Divergent	Tandem	Convergent
Biofilm formation	3	6	0
Carbohydrate metabolic process	2	3	1
Cell adhesion	0	2	0
Cell cycle	17	29	8
Cell development	1	1	0
Cellular homeostasis	3	5	2
Cellular protein modification process	15	38	16
Cellular respiration	1	1	0
Cytokinesis	0	2	0
Cytoskeleton organization	5	4	4
DNA metabolic process	9	11	5
Filamentous growth	20	40	15
Generation of precursor metabolites and energy	1	2	1
Growth of unicellular organism as a thread of attached cells	0	2	0
Hyphal growth	0	2	0
Interspecies interaction between organisms	6	12	5
Lipid metabolic process	4	14	1
Nucleus organization	0	1	0
Organelle organization	62	89	52
Pathogenesis	7	9	5
Protein catabolic process	1	4	3
Pseudohyphal growth	0	1	0
Regulation of biological process	87	180	94
Response to chemical	22	63	26
Response to drug	4	20	4
Response to stress	30	66	33
Ribosome biogenesis	6	6	2
RNA metabolic process	50	43	27
Signal transduction	5	1	2
Translation	5	8	6
Transport	16	46	27
Vesicle-mediated transport	5	8	3
Vitamin metabolic process	2	1	1

## Data Availability

Complete information on the Datasets utilized for analysis is provided in the Methods section of this manuscript.
